# Effect of Mg-Modified Waste Straw Biochar on the Chemical and Biological Properties of Acidic Soils

**DOI:** 10.3390/molecules28135225

**Published:** 2023-07-05

**Authors:** Zhigao Liu, Di Yuan, Xianxian Qin, Peng He, Yunlin Fu

**Affiliations:** 1 College of Resources, Environment and Materials, Guangxi University, Nanning 530004, China; liu_zhi_gao@163.com (Z.L.); yuandi1010@163.com (D.Y.); 2 State Key Laboratory of Featured Metal Materials and Life-Cycle Safety for Composite Structures, MOE Key Laboratory of New Processing Technology for Nonferrous Metals and Materials, Nanning 530004, China; 3 College of Forestry, Guangxi University, Nanning 530004, China; gxulxyqxx@sina.com (X.Q.); hepeng9536@163.com (P.H.)

**Keywords:** biochar, acidic soils, chemical properties, indoor soil simulation experiment, Mg-modified

## Abstract

Biochar is important for soil improvement, fertilizer innovation, and greenhouse gas reduction. In this paper, Mg-modified biochar was prepared from rice and corn straw and mixed with soil at a 1% (*w*/*w*) addition in an indoor soil simulation experiment to study the effect of Mg-modified biochar on the chemical properties of acidic soil. The results showed that the addition of Mg-modified biochar reduced soil acidity and improved soil fertility. Compared with the control group, the Mg-modified biochar treatment significantly increased the concentrations of available potassium, available phosphorus, total phosphorus, organic carbon and exchangeable calcium and magnesium in the soil, and effectively increased the concentration of total nitrogen. Rice straw Mg-modified biochar treatment was more effective in increasing the soil-available potassium, available phosphorus, total phosphorus and exchangeable magnesium concentration, while corn straw Mg-modified biochar was more effective in increasing the soil organic carbon and exchangeable calcium concentration. In addition, the high pyrolysis temperature of Mg-modified biochar was more effective in promoting the soil-available potassium, available phosphorus and total nitrogen concentration, while the low pyrolysis temperature of Mg-modified biochar was more effective in promoting soil alkaline nitrogen, exchangeable calcium and magnesium.

## 1. Introduction

Soil acidification is one of the manifestations of soil degradation [1]. Worldwide, acidic soils are found primarily in tropical, subtropical, and temperate regions. Soil acidification can cause serious harm to agroforestry production and the ecological environment [2]. Acidification also can activate and release aluminium from the soil solid phase into the soil solution, which can be toxic to crop roots and affect crop growth [3]. In addition, soil acidification can accelerate the loss of soil nutrients and reduce soil fertility, as well as increase the hazard of heavy metal pollution [4]. Currently, both rice and corn straw resources in China are directly burnt and not effectively utilised. Biochar is prepared by highly aromatising the structure and stability of organic waste such as crop straw, poultry manure and municipal sludge under high-temperature and anoxic conditions, which not only reduces environmental pollution but also makes comprehensive use of resources [5,6]. Numerous studies in recent years have shown that the application of biochar can enrich mineral nutrients and improve the physical, chemical and biological properties of soils [7,8,9]. The conversion of waste biomass into biochar could help to mitigate CO_2_ and CH_4_ emissions and increase carbon sequestration in the soil for sustainable climate-smart agriculture. The use of biochar in soils is therefore widely recommended for a variety of reasons related to availability and sustainability.

Biochar has a liming effect, which increases the pH of the soil [10]. In addition, biochar can increase soil microbial activity during application by increasing carbon [11], affecting microbial biomass [12] and phosphatase activity [13]. Recently, biochar has been considered an effective organic amendment for acidic soils [14]. Modified biochar usually has a larger specific surface area, a rich pore structure and functional groups. It was found that the chemical activation of biochar is also strengthened by the addition of magnesium salts [15], and the application of MgO-biochar increased the available P concentration of the soil, resulting in higher rice yields [16]. Moreover, sulphur- and iron-modified biochar can improve the pH and organic matter concentration of the soil [17].

In general, biochar nutrients such as P, K, Ca, surface area and pH increase, while the concentration of dissolved organic matter decreases when the temperature of biochar production increases [18]. As the properties of biochar vary depending on the type of raw material, pyrolysis temperature and production technology [19], biochar can have different effects on soil properties [13,20]. Furthermore, the extent to which biochar affects soil properties is highly dependent on the feedstock and pyrolysis conditions, indicating that different preparation parameters often have significant differences, so experimental conditions limit its general applicability. Magnesium is an important constituent of plant chlorophyll and plays an important role in plant growth. The effective magnesium [21] in China’s soils is in a serious deficiency or deficient state, accounting for approximately 21% of the soil area [22]. The low effective Mg concentration of soils in Guangxi Province is likely due to severe soil acidification and the lack of timely application of Mg fertilizers [22]. Mg-modified biochar was found to be alkaline with a large specific surface area and a rich pore structure [23]. There are few studies on the changes in chemical properties of acidic soils over time with Mg-modified biochar, and the effect of Mg-modified biochar with different pyrolysis temperatures on acidic soils is not well understood. Therefore, the use of Mg-modified biochar with different pyrolysis temperatures on acidic soils is necessary and valuable.

The soils in Guangxi are predominantly acidic, and forest trees have a high demand for sustainable soil fertility, so it is crucial to explore a green and efficient soil amendment. Based on the above, indoor short-term soil simulation incubation experiments were used in this paper to investigate the changes in the chemical and biological properties of acidic soil by Mg-modified biomass charcoal from rice and corn straw over time, and to explore the mechanism of the effect of Mg-modified biomass charcoal on soil properties at different pyrolysis temperatures, with the aim of providing a theoretical basis for the improvement of soil chemical and biological properties in forest land by Mg-modified biochar.

## 2. Results

### 2.1. Effect on Soil pH

**Figure 1:**
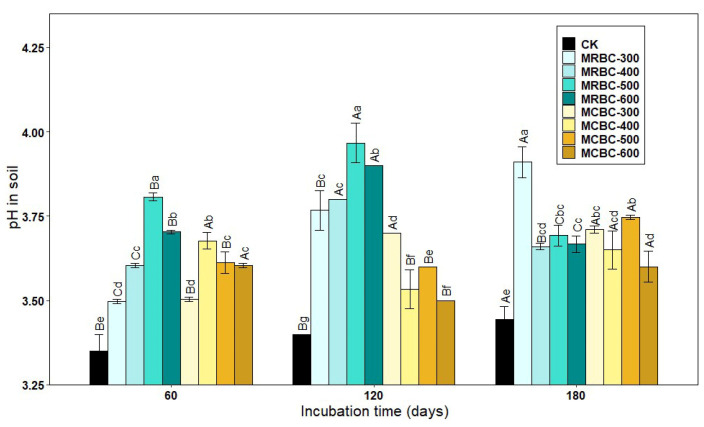
Effect of different Mg-modified biochar on soil pH. Note: The bars in the graph are the mean ± standard deviation of the indicators; lowercase letters indicate the variability of the effect of different Mg-modified biochar on soil chemical properties at the same incubation time (*p* < 0.05) and uppercase letters indicate the variability of the same treatment at different incubation times (*p* < 0.05), as below.

Figure 1 shows the effect of soil pH over time for different Mg-modified biochar treatments. As shown in Figure 1, the soil pH of the Mg-modified biochar treatment was significantly (*p* < 0.05) higher than that of the control group. At 60 and 120 days of incubation, the soil pH of MRBC (Mg-modified Rice straw Biochar) tended to increase and then decrease as the pyrolysis temperature increased, with the MRBC-500 treatment having the best effect on soil acidity improvement. Moreover, the effect of MRBCs on soil pH was better than that of MCBCs at this incubation time, and there was no better regularity in the effect of MCBCs on the pH of the soil. At day 180 of incubation, it was obvious that the MRBC-300 treatment had a significantly higher pH than the control.

With increasing incubation time, the pH of soil treated with MRBCs showed a significant increase at first and then decreased, while the pH value of soil treated with MCBC (Mg-modified Corn straw Biochar) showed an increasing trend. On day 180, the pH value of soil treated with MRBCs increased by 39.2%, 50.6%, 31.0% and 32.1% compared with the initial biochar addition, while the pH value of soil treated with MCBCs and the control group increased by 2.8%, 18.3%, 31.1%, 28.7% and 35.6%, respectively.

### 2.2. Effect on Soil-Available Potassium Concentration

**Figure 2:**
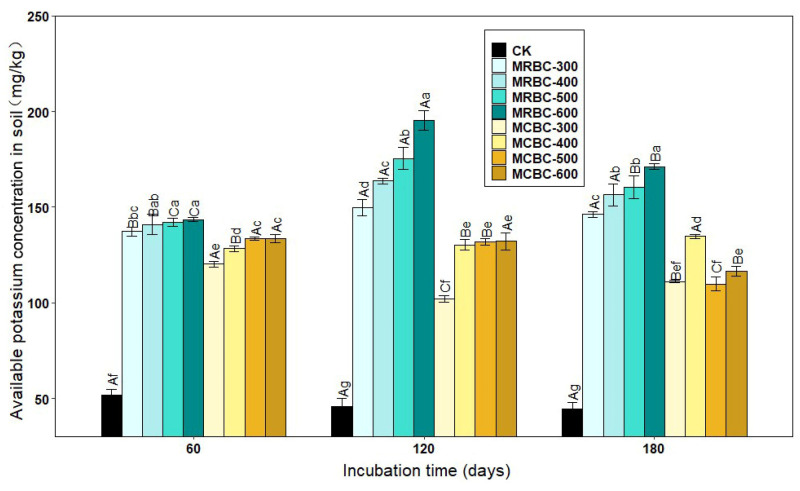
Effect of different Mg-modified biochar on soil-available potassium. Note: The bars in the graph are the mean ± standard deviation of the indicators; lowercase letters indicate the variability of the effect of different Mg-modified biochar on soil chemical properties at the same incubation time (*p* < 0.05) and uppercase letters indicate the variability of the same treatment at different incubation times (*p* < 0.05), as below.

The form of available potassium is easily absorbed and used by plants. As shown in Figure 2, there was a significant (*p* < 0.05) increase in the available potassium concentration of the soil treated with Mg-modified biochar compared to the control group. Overall, the available potassium concentration of the Mg-modified biochar treatments all showed an increasing trend as the biochar pyrolysis temperature increased. When the pyrolysis temperatures of the two straw Mg-modified biochar treatments were the same, the potassium concentration of the MRBC treatment was significantly higher than that of the MCBC treatment.

At day 60, the available potassium concentration of the MRBC treatments was essentially the same for the different pyrolysis temperatures, while the available potassium concentration of the MCBC treatments increased slightly with pyrolysis temperature. On days 120 and 180 of incubation, the available potassium concentration of the MRBC treatment increased significantly with an increasing pyrolysis temperature. On day 180 of incubation, the available potassium concentration of the MCBC-400 treatment was significantly higher than that of the MCBC-300 treatment. The available potassium concentration of the MCBC treatment tended to decrease when the biochar pyrolysis temperature was between 400 °C and 600 °C. The treatment with the best increase in the soil-available potassium concentration at all three incubation time points was MRBC-600 with 177.0%, 328.7% and 283.2% available potassium concentration, respectively, which was significantly higher than the control. The MRBC treatments all showed increasing and then decreasing trends in the soil-available potassium concentration with increasing incubation time, while MCBC-300, MCBC-500 and MCBC-600 treatments showed a decreasing trend in the available potassium concentration with an increasing incubation time. In general, MRBCs were more effective than MCBCs at increasing the available soil potassium concentration.

### 2.3. Effect on Soil-Available Phosphorus Concentration

**Figure 3:**
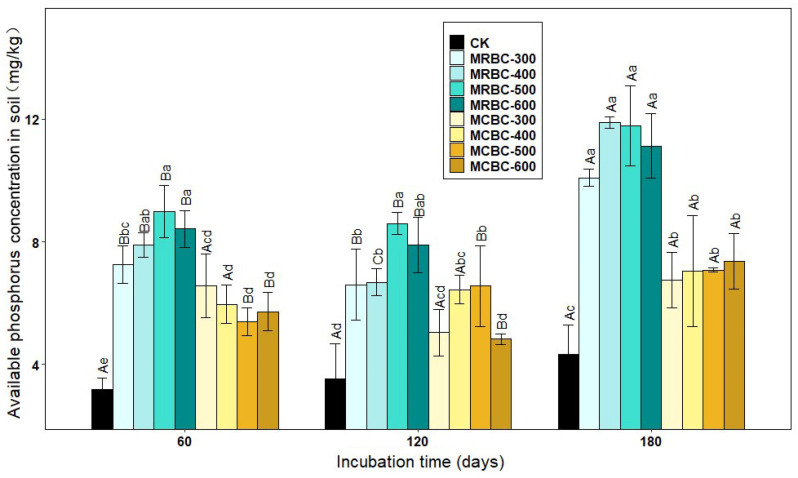
Effect of different Mg-modified biochar on soil-available phosphorus. Note: The bars in the graph are the mean ± standard deviation of the indicators; lowercase letters indicate the variability of the effect of different Mg-modified biochar on soil chemical properties at the same incubation time (*p* < 0.05) and uppercase letters indicate the variability of the same treatment at different incubation times (*p* < 0.05), as below.

The form of available phosphorus is more readily absorbed and used by plants and is the most effective part of the soil’s effective phosphorus reservoir for crops. As shown in Figure 3, the addition of Mg-modified biochar increased the soil-available phosphorus concentration in the treatments compared to the control. Most of the MRBC treatments had significantly (*p* < 0.05) higher soil-available phosphorus concentrations than the MCBC treatments at the same pyrolysis temperature.

At day 60 of incubation, the available phosphorus concentration of the MRBC treatment tended to increase as the biochar pyrolysis temperature increased and, conversely, the available phosphorus concentration of the MCBC treatment tended to decrease, and the differences between the MCBC treatments were small. At day 120 of incubation, the available phosphorus concentrations of the MCBC treatments were higher than that of the control, and all treatments were significantly higher than the control except for MCBC-300 and MCBC-600, which were slightly higher than the control. At day 180 of incubation, the differences in the soil-available phosphorus concentration between treatments with the same straw biochar prepared at different pyrolysis temperatures did not reach significance, but the available phosphorus concentration of MRBC treatment was significantly higher than that of MCBCs. At the three incubation time points, the treatments with the best effect on increasing the soil-available phosphorus concentration were MRBC-500, MRBC-500 and MRBC-400, respectively, which were higher than that of the control group by 181.8%, 143.0% and 174.9%, respectively. At day 180, the available phosphorus concentration was significantly higher in the MRBC treatments than at days 60 and 120. The highest values of available phosphorus for all MRBC treatments occurred at day 180, with MRBCs being more effective at increasing available phosphorus in the soil.

### 2.4. Effect on Soil Alkali-Hydrolysed Nitrogen Concentration

**Figure 4:**
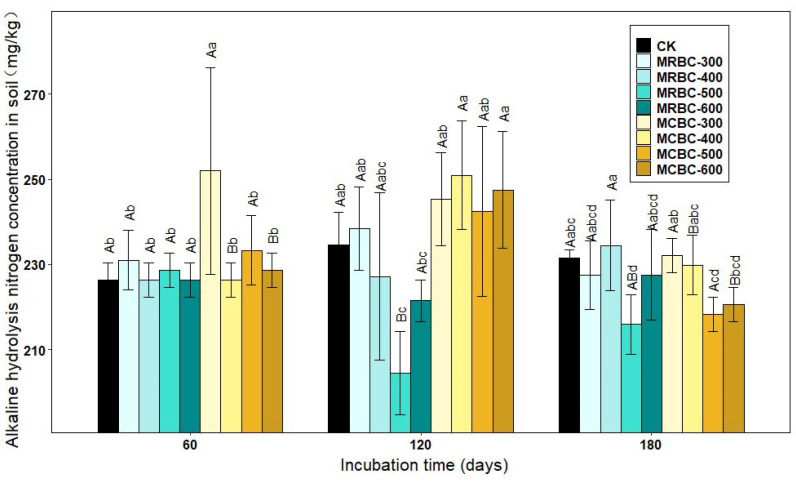
Effect of different Mg-modified biochar on soil alkaline hydrolysis nitrogen. Note: The bars in the graph are the mean ± standard deviation of the indicators; lowercase letters indicate the variability of the effect of different Mg-modified biochar on soil chemical properties at the same incubation time (*p* < 0.05) and uppercase letters indicate the variability of the same treatment at different incubation times (*p* < 0.05), as below.

Figure 4 shows the effect of Mg-modified biochar treatments on the soil alkaline nitrogen concentration. At day 60, the soil alkaline nitrogen concentration of the MCBC-300 treatment was significantly higher than the other treatments (*p* < 0.05) and significantly higher than the control by 11.3%. At day 120 of incubation, the MRBC-300 and MCBC treatments had slightly higher alkaline nitrogen concentrations than the control. At day 180, the Mg-modified biochar was not effective in improving the alkaline nitrogen concentration. The treatments with the best improvement in the soil alkaline nitrogen concentration at the three incubation time points were MCBC-300, MCBC-400 and MRBC-400. Moreover, the soil alkaline nitrogen concentration of each treatment changed less with an increasing incubation time. In summary, it can be seen that the addition of Mg-modified biochar was not effective in promoting the soil alkaline nitrogen concentration and even reduced it in some treatments.

### 2.5. Effect on Total Soil Nitrogen Concentration

**Figure 5:**
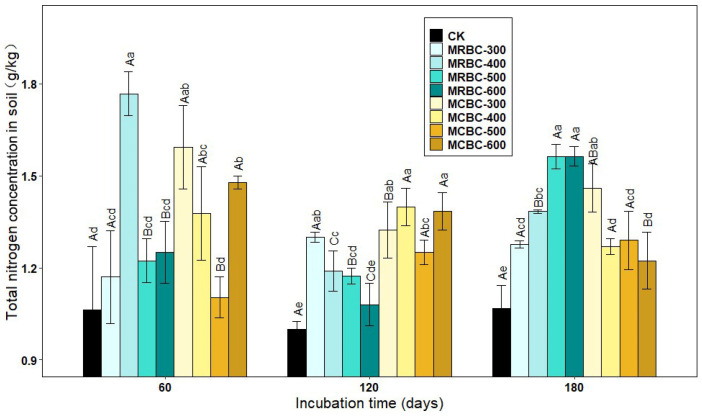
Effect of different Mg-modified biochar on soil total nitrogen. Note: The bars in the graph are the mean ± standard deviation of the indicators; lowercase letters indicate the variability of the effect of different Mg-modified biochar on soil chemical properties at the same incubation time (*p* < 0.05) and uppercase letters indicate the variability of the same treatment at different incubation times (*p* < 0.05), as below.

In addition to available nutrients, biochar also had an effect on total soil nutrients. As shown in Figure 5, the addition of Mg-modified biochar treatments increased the soil total nitrogen concentration compared to the control. At day 60, the soil total nitrogen concentration of MRBC-400, MCBC-300, MCBC-400 and MCBC-600 treatments were significantly higher than that of the control by 66.1%, 49.8%, 29.5% and 38.9%, respectively, with MRBC-400 being the highest in each treatment, followed by MCBC-300. At day 120, the soil total nitrogen concentration was significantly higher in each treatment, except the MRBC-600 treatment, than in the control. Furthermore, as the temperature of biochar pyrolysis increased, the total nitrogen concentration of the MRBC treatments became progressively lower, with the MRBC-300 treatment having a significantly higher total nitrogen concentration than the control and other MRBC treatments. It is clear that the difference in the total nitrogen concentration between the treatments of MCBCs was small and lacked any significant regularity. At day 180, the total nitrogen concentration of MRBCs gradually increased as the temperature of biochar pyrolysis increased, and the MRBC treatments with high-temperature pyrolysis (500 and 600 °C) were significantly higher than those with low-temperature pyrolysis (300 and 400 °C). Conversely, the total nitrogen concentration of the MCBC treatments tended to decrease, with the MCBC-300 treatment having a significantly higher total nitrogen concentration than the other treatments. As the incubation time increased, MRBC-400, MRBC-500 and MRBC-600 treatments all showed a trend of decreasing and then increasing, while there was some variation but no regularity in the total nitrogen concentration of the MCBC treatments at each incubation time.

### 2.6. Effect on Soil Total Phosphorus Concentration

**Figure 6:**
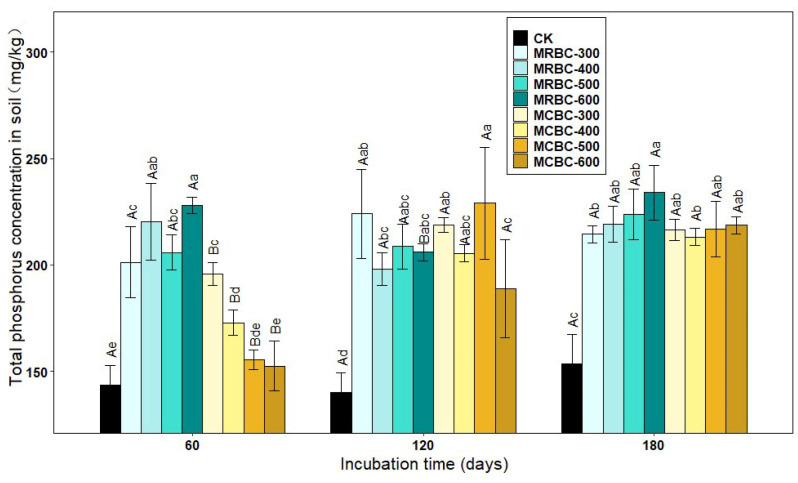
Effect of different Mg-modified biochar on soil total phosphorus. Note: The bars in the graph are the mean ± standard deviation of the indicators; lowercase letters indicate the variability of the effect of different Mg-modified biochar on soil chemical properties at the same incubation time (*p* < 0.05) and uppercase letters indicate the variability of the same treatment at different incubation times (*p* < 0.05), as below.

As shown in Figure 6, the treatments with the addition of Mg-modified biochar showed an increase in the soil total phosphorus concentration compared to the control. At day 60, the total phosphorus concentration of the soil was significantly (*p* < 0.05) higher in the MRBC treatment than in the control and showed an overall increasing trend with increasing biochar pyrolysis temperature in the MRBC treatment, but a decreasing trend in the MCBC treatment. It was clear that the MRBC treatment had a higher total phosphorus concentration than the MCBCs, with the MRBC-600 treatment being the best and having a significantly higher total phosphorus concentration than the control at 58.8%, while the MCBC-300 treatment had a significantly higher total phosphorus concentration than the control at 36.5%. At day 120, the total phosphorus concentration of the soil was significantly higher in the MCBC treatment than in the control, but the differences between the treatments were small. At day 180, the soil total phosphorus concentration was significantly higher in each treatment than in the control, but there was no significant change between treatments. As the temperature of biochar pyrolysis increased, the soil total phosphorus concentration of the MRBC treatments gradually increased, with the MRBC-600 treatment being significantly higher than the MRBC-300 treatment. At the three incubation time points, the treatments with the best effect on increasing the soil total phosphorus concentration were MRBC-600, MCBC-500 and MRBC-600, which were 58.8%, 63.4% and 52.5% higher than the control, respectively.

The total phosphorus concentration of the MRBC treatment tended to increase with incubation time, but the increase was small. There was no uniform pattern of change in the total phosphorus concentration of the MCBC treatments, but at day 180, the total phosphorus concentration of the soil tended to be the same for all treatments.

### 2.7. Effect on Soil Organic Carbon Concentration

**Figure 7:**
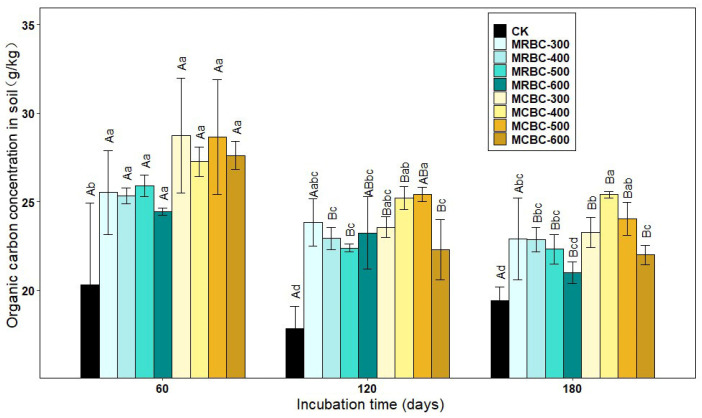
Effect of different Mg-modified biochar on soil organic carbon. Note: The bars in the graph are the mean ± standard deviation of the indicators; lowercase letters indicate the variability of the effect of different Mg-modified biochar on soil chemical properties at the same incubation time (*p* < 0.05) and uppercase letters indicate the variability of the same treatment at different incubation times (*p* < 0.05), as below.

As shown in Figure 7, the soil organic carbon concentration increased significantly (*p* < 0.05) in the Mg-modified biochar treatment compared to the control group. At day 60, soil organic carbon concentration was greater in the MCBC treatments than in the MRBCs. At day 120, the organic carbon in the MRBC treatment tended to decrease and then increase as the biochar pyrolysis temperature increased, while the MCBC treatment tended to increase and then decrease. Moreover, the differences in organic carbon concentration between the MRBC treatments were small, while the organic carbon concentration of the MCBC-400 and MCBC-500 treatments was significantly greater than that of MCBC-600. At day 180, the organic carbon of MRBC treatment showed a decreasing trend as the biochar pyrolysis temperature increased. Moreover, as the biochar pyrolysis temperature varied from 400 to 600 °C, the organic carbon concentration of the MCBC treatments decreased, and it could be found that the organic carbon concentration of the MCBC-400 treatment was significantly higher than that of MCBC-300 and MCBC-600. At the three incubation time points, the treatments with the best effect on increasing the soil organic carbon concentration were MCBC-300, MCBC-500 and MCBC-400, which were significantly higher than the control by 41.5%, 42.3% and 30.9%, respectively. As the incubation time changed, the soil organic carbon concentration of all treatments tended to decrease gradually.

### 2.8. Effect on Soil Exchangeable Calcium Concentration

**Figure 8:**
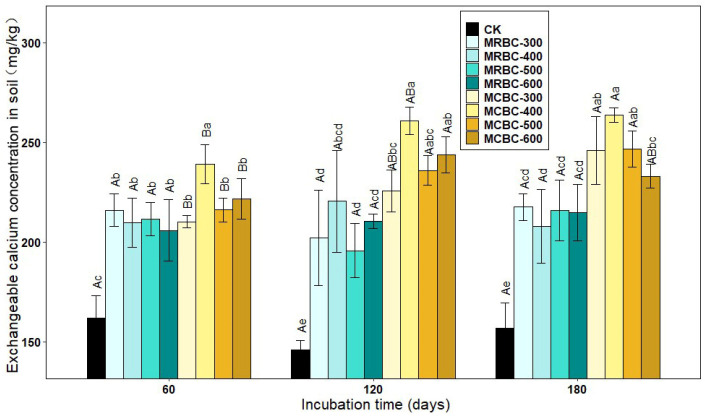
Effect of different Mg-modified biochar on soil exchangeable calcium. Note: The bars in the graph are the mean ± standard deviation of the indicators; lowercase letters indicate the variability of the effect of different Mg-modified biochar on soil chemical properties at the same incubation time (*p* < 0.05) and uppercase letters indicate the variability of the same treatment at different incubation times (*p* < 0.05), as below.

As shown in Figure 8, the exchangeable calcium concentration was significantly (*p* < 0.05) increased in the Mg-modified biochar treatment compared to the control. The differences in the exchangeable calcium concentration between the MRBC treatments were lower between the various incubation time periods. At day 60, among the MCBC treatments, the highest exchangeable calcium concentration was found in MCBC-400 for each treatment and was significantly different from the other treatments, with less difference in the exchangeable calcium concentration between the other treatments. At day 120, the differences in the exchangeable calcium concentration between the MCBC-400, MCBC-500 and MCBC-600 treatments were not significant, and the MCBC-400 treatment was significantly higher than MCBC-300 and the control. At day 180, among the MCBC treatments, the exchangeable calcium concentration was significantly higher in the MCBC-400 treatment than in the MCBC-600 and control groups, with no significant differences between the other treatments. The MCBC treatment had higher exchangeable calcium than the MRBCs at the same pyrolysis temperature for both straw-Mg modified biochar.

At all three incubation time points, the treatment with the best effect on increasing the soil exchangeable calcium concentration was MCBC-400, which was significantly higher than the control by 49.5%, 78.5% and 65.1%, respectively. As the incubation time increased, the MCBC treatment showed an increasing trend in the exchangeable calcium concentration, but the increase was smaller.

### 2.9. Effect on Exchangeable Magnesium Concentration in Soil

**Figure 9:**
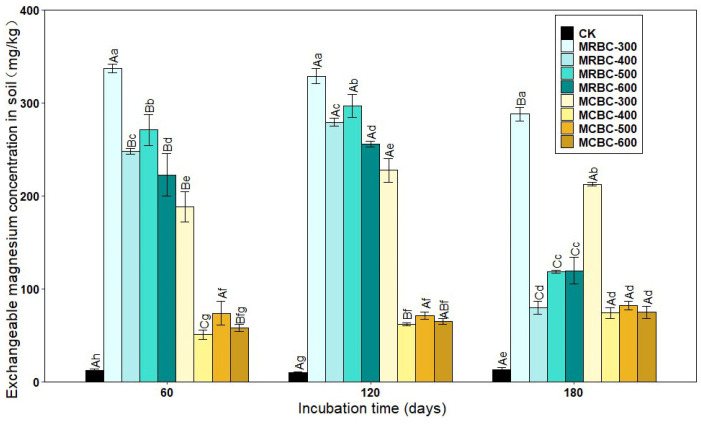
Effect of different Mg-modified biochar on soil exchangeable magnesium. Note: The bars in the graph are the mean ± standard deviation of the indicators; lowercase letters indicate the variability of the effect of different Mg-modified biochar on soil chemical properties at the same incubation time (*p* < 0.05) and uppercase letters indicate the variability of the same treatment at different incubation times (*p* < 0.05), as below.

Exchangeable magnesium is magnesium that is adsorbed by soil colloids and can be exchanged by general exchange agents and is available to plants. As shown in Figure 9, the Mg-modified biochar treatment significantly (*p* < 0.05) increased the soil exchangeable magnesium concentration when compared to the control. As the pyrolysis temperature increased, the soil exchangeable Mg concentration of the MRBC treatment tended to decrease. At all three incubation time points, the differences in the exchangeable Mg concentration between the MCBC-400, MCBC-500 and MCBC-600 treatments were small, and all three treatments were significantly lower than the MCBC-300 treatment. Particularly, the treatment with the best amelioration of the soil exchangeable Mg concentration at different time periods was MRBC-300, which was higher than the controls by 2570.2%, 3061.1% and 2089.2%, respectively.

As the incubation time increased, the soil exchangeable Mg concentration of the MRBC treatment tended to increase before significantly decreasing, whereas the MCBC treatments all tended to increase the exchangeable Mg concentration. At day 180, the MRBC treatment showed a decrease of 14.6%, 67.7%, 56.2% and 46.2%, respectively, compared to the initial biochar addition, while the MCBC treatment showed an increase of 13.0%, 46.0%, 10.9% and 28.7%, respectively.

### 2.10. Effect on Soil Cation Exchange Capacity

**Figure 10:**
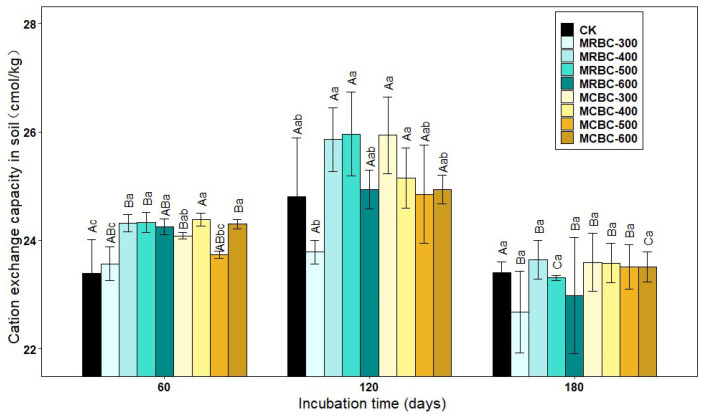
Effect of different Mg-modified biochar on soil cation exchangeable capacity. Note: The bars in the graph are the mean ± standard deviation of the indicators; lowercase letters indicate the variability of the effect of different Mg-modified biochar on soil chemical properties at the same incubation time (*p* < 0.05) and uppercase letters indicate the variability of the same treatment at different incubation times (*p* < 0.05), as below.

Soil cation exchange capacity (CEC) is the total amount of various cations that can be adsorbed by the soil colloids. As can be seen from Figure 10, at day 60, soil CEC was higher in all treatment groups, except MRBC-300 and MCBC-500, than in the control group. At day 120, the soil CEC of MRBC400, MRBC-500 and MCBC-300 treatments were significantly higher than the other treatment groups, while there was no significant difference in the soil CEC of the biochar treatments at day 180. As the incubation time increased, the CEC of each treatment showed a trend of increasing and then decreasing, with an overall decreasing trend. At day 60, some of the Mg-modified biochar treatments had an increasing effect on the CEC, but in the latter two stages, the promotion effect gradually decreased and there was no significant difference between the treatments and the control group.

### 2.11. Effect on Soil Microbial Biomass Carbon and Microbial Biomass Nitrogen

**Figure 11:**
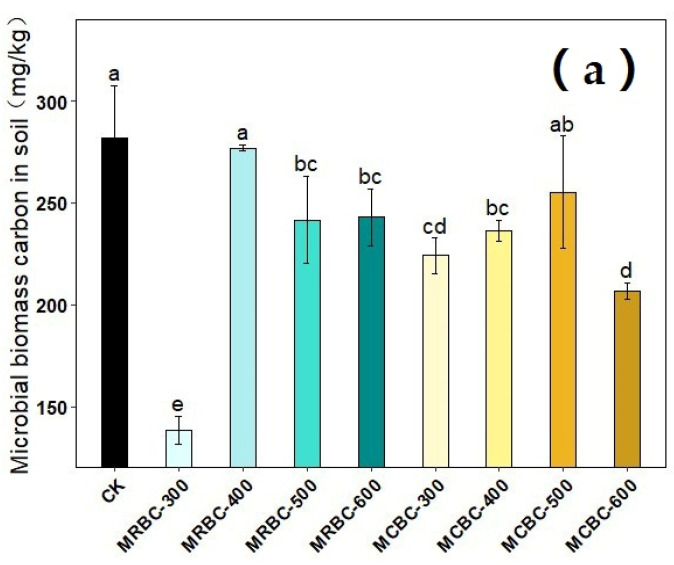

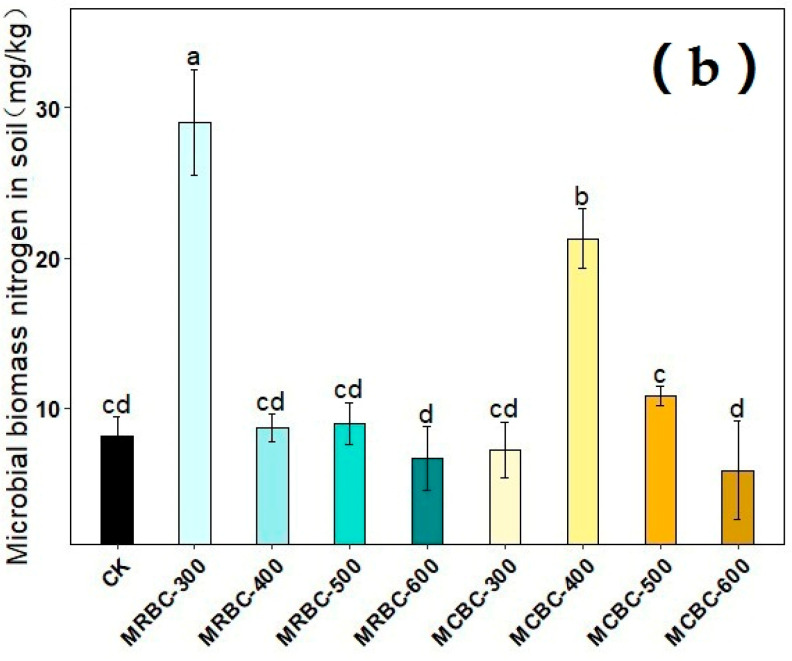
Effect of different Mg-modified biochar on soil microbial biomass carbon (**a**) and nitrogen (**b**). Note: The bars in the graph are the mean ± standard deviation of the indicators; lowercase letters indicate the variability of the effect of different Mg-modified biochar on soil chemical properties at the same incubation time (*p* < 0.05).

The effects of different pyrolysis temperature Mg-modified biochar treatments on soil microbial biomass carbon and microbial biomass nitrogen are shown in Figure 11. On day 180 of soil incubation, the soil microbial biomass carbon content was reduced in all MRBCs and MCBC treatments compared to the control. Meanwhile, only the MRBC-300 and MCBC-400 treatments (Figure 11b) showed an increase in the soil microbial biomass nitrogen content, while the other treatments were not significantly different from the control. The treatment with the best effect on the increase in the soil microbial biomass nitrogen content was MRBC-300 and was significantly higher than the control by 256.2%.

## 3. Discussion

A meta-analysis showed that the combined effect of biochar and soil had a large impact on plant growth, regardless of the nature of the biochar and soil conditions, and that the effect of biochar on plant productivity was estimated to be 16.0~1.26% [24], making the addition of biochar to soil an important methods of soil fertility management. In this study, the addition of Mg-modified biochar from rice and corn straw increased soil pH and increased the concentration of available phosphorus, available potassium, alkaline digested nitrogen and total nitrogen. This may be due to the alkaline nature of biochar with its high cation exchange capacity and own nutrient concentration and effectiveness, and the presence of functional groups such as carboxyl groups and alkalinity on the surface [25], which determine the effectiveness of biochar in improving soil fertility [26].

Acidic soils account for 40% of the world’s potentially arable land and affect plant growth to some extent. This experiment showed that the addition of Mg-modified biochar from rice and corn straw increased soil pH, and that this increased with an increasing biochar pyrolysis temperature. This is because the basic groups of Mg-modified biochar increase and the acidic groups decrease with an increasing pyrolysis temperature [23,27]. The oxygen-limited pyrolysis of Mg-modified biochar contains a variety of basic substances such as carbonate (CaCO_3_), carbonyl (COO^−^) and phosphate (PO_4_^3−^) [28]. The alkaline material in Mg-modified biochar neutralised the soil acidity and increased soil pH when applied to acidic soils [29], and high-temperature cracked biochar was generally higher than low-temperature cracked biochar for soil pH [30], which is consistent with the results of other studies [31]. In this study, the pH of the treatment with the addition of Mg-modified biochar from rice straw showed an increasing trend followed by a decreasing trend, which may be due to the continuous release of cations contained in the biochar during the decomposition of soil microorganisms by Mg-modified biochar, which absorbs and holds exchangeable cations and aluminium ions in the soil [32]. The pH of the treatment with Mg-modified biochar from corn straw showed an increasing trend with increasing incubation time, but this was likely due to the slow release of alkaline substances from the Mg-modified corn straw biochar in the soil to neutralise soil acidity. Overall, the Mg-modified corn straw biochar was more effective at improving the acidic soil. Although biochar had a significant effect on soil pH in this study, the amount of alkaline material in biochar is usually limited, the increase in soil pH is not sustainable and soil pH only increases when biochar application rates are high (> 1–5%) [32].

In this study, the soil-available potassium concentration increased with an increasing Mg-modified biochar pyrolysis temperature. Elemental K, a common mineral element in the ash of biochar, increased with an increasing Mg-modified biochar pyrolysis temperature [33], and higher pyrolysis temperatures of Mg-modified biochar were more effective at increasing the available potassium concentration of the soil. In the crystal structure of Mg-modified biochar in the previous paper, a diffraction peak of KCl crystals appeared when the pyrolysis temperature was 600 °C, producing potassium salts at high pyrolysis temperatures [23]. In addition, Mg-modified biochar can also indirectly increase the available potassium concentration by changing the soil pH or the amount of cation exchange.

In this experiment, the addition of Mg-modified biochar resulted in a significant increase in the available phosphorus and total phosphorus concentration of the soil. The phosphorus adsorbed on biochar can be slowly released for plant growth through the adsorption–desorption equilibrium [34], and the results of this study are consistent with those of other experiments [35]. Furthermore, in acidic soils, where soil phosphorus is primarily constrained by the hydroxides or oxides of iron and aluminium, the neutralising effect of biochar on the soil helps to increase the effectiveness of the available phosphorus [36]. It may also be because biochar, due to its strong cation exchange capacity, can effectively increase soil phosphorus effectiveness by providing a negative surface charge or by influencing elements such as iron and aluminium that bind to phosphorus [37].

Nitrogen is essential for plant nucleic acids and proteins and affects plant photosynthesis. The addition of biochar can directly influence the soil N cycle and reduce N efflux [38]. The effect of biochar on the soil inorganic nitrogen concentration is influenced by the biochar material, pyrolysis temperature, application time and soil type [39]. In the present study, the addition of Mg-modified biochar increased the soil total N concentration. This may be because biochar contains a certain amount of nitrogen, which can be directly increased by the addition of biochar, or because the microbial immobilisation effect enhances the retention of nitrogen or fertiliser nitrogen in the soil [40]. The difference between the Mg-modified biochar treatments and the control on soil alkali-dissolved nitrogen was not significant, and the increase in the alkali-dissolved nitrogen concentration in some of the treatments may be due to biochar accelerating the mineralisation of soil organic-state nitrogen and releasing more inorganic-state nitrogen [41]. The change in the alkali-dissolved nitrogen concentration with incubation time was small for each biochar treatment, which is similar to the results of Ramlow et al. [42].

Organic carbon is essential for microorganisms, plants and animals and is a key carbon source for soil microorganisms, which are vital for maintaining soil fertility and long-term agricultural sustainability. An increased soil organic carbon concentration can improve the condition of soil aggregates and enhance soil fertility retention. In this study, the addition of Mg-modified biochar significantly increased the soil organic carbon concentration due to the stable structure within the biochar inhibiting the surface oxidation of organic carbon, which reduces the mineralisation rate of organic carbon and therefore increases the organic carbon concentration [43]. Secondly, biochar, a stable organic carbon fraction, directly increases the organic carbon concentration through physical mixing with the soil, changing the composition of soil organic carbon [16]. According to previous studies, it is known that Mg-modified biochar has a high C (%) concentration, H/C and O/C, and a high aromaticity, indicating that the biochar is rich in C elements and has a more stable structure [23,27]. As the incubation time changed from 60 to 120 days, the organic carbon concentration of each treatment decreased, which was due to the decomposition and utilisation by soil microorganisms. As the incubation time changed from 120 to 180 days, the organic carbon concentration of each biochar treatment changed less, likely because most of the carbon in biochar at this time belonged to the aromatic stable state of carbon that was difficult to oxidise and utilise, and was more stable, making it difficult for microorganisms to decompose its organic carbon fraction [44].

In this study, the addition of Mg-modified biochar significantly increased the exchangeable calcium and magnesium concentration of the soil. Based on previous studies, it is found that Mg is primarily present as Mg_2_SiO_4_ and Mg(OH)_2_ in Mg-modified biochar, therefore the addition of Mg-modified biochar can directly increase the exchangeable Mg concentration in the soil [23,27]. Secondly, Mg-modified biochar is characterised by large pores, high negative surface charge and high specific surface area, which can adsorb and retain cations (e.g., Mg^2+^, Ca^2+^, K^+^ and NH^4+^) in the soil [45], which is similar to the results of previous studies [46]. In addition, an increase in soil pH also increases the soil exchangeable calcium and magnesium concentration [47]. Soil nutrient status was classified into five levels according to the recommended fertilisation indexes of the systematic study method, i.e., less than 60 mg/L of soil-effective Mg is severely deficient, 60–120 mg/L is deficient, 120–300 mg/L is moderate, 300–600 mg/L is abundant and greater than 600 mg/L is very abundant [48]. Comparing these levels, in this study, the control soil Mg concentration was severely deficient, and most of the soil effective Mg concentration levels after the addition of Mg-modified corn straw biochar were deficient; the soil-effective Mg concentration after the addition of Mg-modified rice straw biochar was moderate. This shows that the addition of rice and corn straw Mg-modified biochar can effectively increase the soil-effective calcium and magnesium concentration, and rice straw Mg-modified biochar is more effective at increasing the soil-effective Mg concentration.

Soil cation exchange capacity (CEC) can reflect the fertilizer retention and supply capacity of the soil [49]. In this study, the CEC of both 300 °C and 600 °C biochar-added soils decreased to varying degrees compared to the control group. On the one hand, this may be due to the higher CEC of the test soils in the study and the fact that biochar application increased CEC for soils with lower CEC and decreased slightly for soils with higher CEC [50]. In addition, the type of feedstock and pyrolysis temperature of the biochar also influenced the soil CEC capacity. From Figure 10, it can be seen that rice and corn straw biochar prepared at pyrolysis temperatures of 400 and 500 °C promoted soil CEC better, which is consistent with literature reports [51].

Related studies [52] have shown that mixing rice and corn straw biochar prepared at pyrolysis temperatures of 300 °C, 400 °C and 500 °C at 1% with red loamy rice soils for 135 days in an indoor simulated incubation trial compared to the indoor soil incubation trial (day 120) of rice and corn straw Mg-modified biochar at different pyrolysis temperatures in this paper both significantly increased soil pH. The increase in soil organic carbon and available phosphorus concentration was greater for the Mg-modified biochar compared to the control. In both studies, biochar increased the soil pH and available potassium concentration by similar amounts, and rice straw biochar addition to soil alkaliolytic nitrogen decreased slightly in both studies, but corn straw biochar addition was more effective in increasing soil alkaliolytic nitrogen, while the available potassium concentration varied in a similar pattern with temperature. It can be seen that Mg-modified biochar increased soil organic carbon, available phosphorus and the alkaline nitrogen concentration to a greater extent, indicating that Mg-modified biochar promotes nutrients related to soil C, P and N cycling and has a certain promotion effect on soil chemical properties. This may be due to the increased specific surface area and pore space of the modified biochar, which can better adsorb nutrients and reduce nutrient loss. It can be seen that rice and corn straw biochar have a positive effect on soil physicochemical properties, whether modified or not, but Mg-modified straw biochar has a better overall effect on soil chemistry. Related studies [53] found that the application of hickory shell biochar to sandy loam soils not only increased soil pH but also increased soil organic carbon, Ca, K, Mn and P concentration, which is similar to the results of this experimental study. However, the results varied from one experiment to another because of the differences in biochar material, soil type, soil fertility and crop species grown. In our earlier work, it was found that corn straw Mg-modified biochar had more carbon and that there was less difference in the nitrogen concentration between the biochar of the two materials [19,27]. Thus, the soil treated with rice straw Mg-modified biochar had a higher pH and faster potassium concentration, the soil treated with corn straw Mg-modified biochar had a higher organic carbon concentration and the difference between the two straw treatments in terms of soil alkaline dissolved nitrogen and total nitrogen concentration was less.

Biochar can influence soil microbial biomass by regulating the microbial living environment and microbial reproduction process. In this experiment, soil microbial biomass carbon was reduced in both MRBCs and MCBC treatments, which may be due to an imbalance in soil microbial metabolism caused by the high total organic carbon content and small changes in total nitrogen after the addition of Mg-modified biochar to the soil, thus inhibiting microbial activity [54]. However, some studies have shown an increase in soil microbial biomass carbon due to biochar application [55], which may be due to differences in the soil type or the physicochemical properties of the biochar. In addition, some of the Mg-modified biochar treatments increased soil microbial biomass nitrogen, possibly because the addition of biochar increased soil nutrient concentrations and provided a habitat for microbial activity, which indirectly increased soil microbial biomass nitrogen.

## 4. Materials and Methods

### 4.1. Preparation of Modified Biochar

In this experiment, rice straw was collected from farmland at the experimental base of the College of Agriculture, Guangxi University, and corn straw was collected from farmland in Mashan County, Guangxi. The samples were collected on 6 November 2019 and 15 November 2019, respectively. The rice and corn stalks were dried in natural air and then crushed with a grinder to obtain a powder that passed through an 80-mesh sieve, and the sieved powder was stored in a sealed bag in a desiccator to be used. The biochar was prepared by oxygen-limited slow cracking. The straw powder was placed in a quartz boat and placed in the heating and constant temperature zone of a tube furnace. Nitrogen was injected into the tube furnace, and the biochar was prepared by high-temperature pyrolysis in the absence of oxygen. It was heated to 300 °C at a heating rate of 8 °C/min and kept at a constant temperature for 1 h. After cooling, the precursor biochar was obtained. This was added to a solution of magnesium chloride at a concentration of 1 mol/L. Every 10 g of precursor biochar was mixed with 500 mL of a 1 mol/L magnesium chloride solution. The mixture was stirred with a constant-temperature magnetic stirrer for approximately 1 h. After vacuum filtration, the precursor biochar was dried in an oven at 106 °C for 6 h before being placed in a tube furnace for pyrolysis. The pyrolysis temperatures were set at 300 °C, 400 °C, 500 °C and 600 °C. The heating rate was also 8 °C/min, and the set temperature was reached and then heated continuously at a constant temperature for 1 h. After cooling and removal, the preparation of Mg-modified biochar from rice and corn straw at different pyrolysis temperatures was completed [23,27]. The prepared Mg-modified biochar was placed in a sealed bag and stored in a desiccator for backup. The names of the Mg-modified biochar are given in Table 1.

### 4.2. Laboratory Simulated Soil Test

#### 4.2.1. Soil Sampling

The soil-sampling site is located in Nanning Arboretum, Guangxi, in the south of Nanning City (108°15′14″~108°22′22″ E, 22°34′31″~22°46′51″ N), which is dominated by low mountainous terrain and has a southern subtropical monsoon climate with abundant water and heat conditions. The soil here is red loam with a thick soil layer at an altitude of approximately 210 m. It is a second-generation forest of *Eucalyptus urophylla* × *E. grandis*, 3 years old, with a plant spacing of 2.0 m × 3.5 m and a vegetation cover of approximately 75%. Soil samples were collected on 4 December 2019. In the plantation of Eucalyptus tailorii, soil samples were taken from 0 to 20 cm in a 20 m × 20 m sample square in an S-shape, and the fresh soil mixture was sieved through a 2 mm sieve and then air-dried and preserved for laboratory-simulated soil tests [53]. The pH and moisture content of the basic soil used in the experiments were 3.43 and 15.63%, respectively. The total nitrogen and phosphorus concentrations of the soil were 1.07 g/kg and 150.2 mg/kg, respectively, while the available phosphorus, alkaline nitrogen and available potassium concentrations were 49.5 mg/kg, 3.35 mg/kg and 205.33 mg/kg, respectively. Additionally, the initial organic matter concentration and cation exchange capacity of the basic soil were 35.89 g/kg and 24.13 cmol/kg, respectively.

#### 4.2.2. Detection of Soil Properties

The Mg-modified biochar was dried for later use. The appropriate amount of biochar addition had a positive effect on the soil chemistry. According to the characteristics and objectives of this study, 1% biochar addition was selected [56]. First, 150 g of air-dried soil was weighed into 500 mL plastic culture bottles, and a total of eight Mg-modified biochar of rice and corn straw mentioned above (MRBC-300, MRBC-400, MRBC-500, MRBC-600, MCBC-300, MCBC-400, MCBC-500, MCBC-600) were mixed thoroughly with the soil. Distilled water was added to 60% of the saturated water volume in the field to maintain some air permeability and then placed in an incubator at (25 ± 1) °C for the incubation experiment. A control group (CK) was set up without any biochar addition. Three replicate groups were set up for each treatment. Soil specimen designations for all treatments were the same as the biochar nomenclature, except for the control group. All soil experimental groups were hydrated by the weighing method at 2-week intervals. The levels of pH, organic matter, available potassium, alkaline soluble nitrogen, available phosphorus, total nitrogen, total phosphorus, exchangeable calcium and magnesium and soil cation exchange capacity were measured every 2 months for 6 months. After the 6th month of the indoor simulated soil test, destructive sampling was carried out and the soil samples were stored under refrigeration at 4 °C to complete the soil microbial biomass carbon and nitrogen concentrations in a short period of time.

The basic properties of the soils were referenced in the method of Rukun Lu [57], and the experimental equipment used in this experiment is shown in Table 2. Soil pH was determined potentiometrically. CEC was determined by leaching with hexaammine-cobalt trichloride. Soil alkaline nitrogen (AN) was determined by the alkaline diffusion method, and soil total nitrogen (TN) was determined by the sulphuric acid-perchloric acid digestion-ammonia electrode method. Soil-available phosphorus (AP) was determined by the sulphuric acid-perchloric acid digestion-atomic spectrometer method, and soil total phosphorus (TP) was determined by the sulphuric acid-perchloric acid digestion-atomic spectrometer method. Soil organic matter was determined by external heating with potassium dichromate. Exchangeable calcium and magnesium were determined by ammonium chloride leaching. Soil microbial biomass carbon and soil microbial biomass nitrogen were determined by chloroform fumigation leaching and flow analyser methods, respectively. Available potassium was determined by ammonium acetate extraction. The data obtained from the above tests were processed in Excel 2016 and then analysed. Differences between soil indicators for different Mg-modified biochar treatments were analysed using SPSS 2.0. Data were first subjected to one-way ANOVA (One-way ANOVA); if *p* < 0.05, there was a significant difference between treatments and further Duncan multiple comparisons were performed. Conversely, if *p* > 0.05, there was no significant difference between treatments. All the figures were constructed using Orgin 8.0 and R 3.6.2 software.

## 5. Conclusions

In this paper, rice and corn straw were selected as raw materials for the preparation of Mg-modified biochar, and the effects of the two types of straw biochar on soil properties were investigated in an indoor soil simulation experiment. The results showed that the Mg-modified biochar treatment of rice and corn straw significantly increased the available potassium, available phosphorus, total phosphorus, organic carbon and exchangeable calcium and magnesium concentrations of the soil. In addition, both MRBCs and MCBC treatments increased soil pH. Furthermore, Mg-modified straw biochar prepared at a higher pyrolysis temperature was more effective at increasing soil pH, available potassium, available phosphorus and the total phosphorus concentration. Conversely, Mg-modified biochar prepared at a lower pyrolysis temperature was more effective at increasing alkaline nitrogen, exchangeable calcium and magnesium concentration. In summary, the MRBC treatments were more effective at increasing soil-available potassium, available phosphorus, total phosphorus and exchangeable magnesium, while the MCBC treatments were more effective at increasing soil-enhanced organic carbon and exchangeable calcium. It can be seen that the addition of MRBCs or MCBCs to acidic soils in certain proportions can effectively improve soil fertility and significantly increase the effective magnesium concentration of the soil. Finally, the addition of Mg-modified biochar resulted in a reduction in soil microbial biomass carbon, but some of the biochar had a beneficial effect on microbial biomass nitrogen. In summary, the appropriate pyrolysis temperature, feedstock and modification or not can be selected according to the different forestry needs.

## Figures and Tables

**Table 1:** Nomenclature of Mg-modified biochar.

Serial Number	The Raw Material	Pyrolysis Temperature (°C)	Continuous Pyrolysis Room (h)	Impregnation Concentration of MgCl_2_ (mol/L)
MRBC-300	Rice straw	300	1	1
MRBC-400	Rice straw	400	1	1
MRBC-500	Rice straw	500	1	1
MRBC-600	Rice straw	600	1	1
MCBC-300	Corn stover	300	1	1
MCBC-400	Corn stover	400	1	1
MCBC-500	Corn stover	500	1	1
MCBC-600	Corn stover	600	1	1

**Table 2:** Test methods for soil properties.

Indicators	Methods	Equipment	Types	Manufacturers
pH value	Potential method	pH meter	pHS-25	Leici Instrument Factory, Shanghai, China
Available potassium	Ammonium acetate extraction method	UV spectrophotometer	UV-2500	Shimadzu Instruments Ltd., Suzhou, China
Available phosphorus	Double acid (HCl-H_2_SO_4_) extraction	UV spectrophotometer	UV-2500	Shimadzu Instruments Ltd., Suzhou, China
Nitrogen alkali digestion	Alkaline diffusion method	UV spectrophotometer	UV-2500	Shimadzu Instruments Ltd., Suzhou, China
Total phosphorus	Sulphuric acid–perchloric acid elimination method	UV spectrophotometer	UV-2500	Shimadzu Instruments Ltd., Suzhou, China
Total nitrogen	Sulfuric acid–perchloric acid elimination method–ammonia electrode	Continuous flow analyzer	PROXIMA	AMS Allinace, Paris, France
Organic matter	High temperature external thermal potassium dichromate oxidation–volumetric method	Enzyme-labeled instrument	INFINITE M200 Pro	Tecan Trading AG, Männedorf, Switzerland
Exchangeable cation	Extraction of cobalt hexanamine trichloride–spectrophotometric method	UV spectrophotometer	UV-2500	Shimadzu Instruments Ltd., Suzhou, China
Exchangeable calcium and magnesium	Atomic absorption spectrophotometry	Flame Atomic Absorption Spectrometer	novAA350	Analytik Jena AG, Jena, German
Microbial biomass carbon	Chloroform fumigation method	Automatic Total Organic Carbon Analyzer	TOC-500	Shimadzu Instruments Ltd., Kyoto, Japan
Microbial biomass nitrogen	Flow analysis method	Continuous flow analyzer	PROXIMA	AMS Allinace, Paris, France
